# Review of Nature and Art in the Cure of Disease

**Published:** 1857-09

**Authors:** John Forbes

**Affiliations:** Fellow of the Royal College of Physicians, Physician to the Queen’s Household, &c., &c.—London


From the British and Foreign Medico-Chirurgical Review.

*Review of Nature and Art in the Cure of Disease.* By Sir JOHN FORBES, M. D., D. C. L. Oxen., F. R. S., Fellow of the Royal College of Physicians, Physician to the Queen's Household, &c., &c.—*London*, 1857. pp. 264.

The inquiry which Sir John Forbes suggests, as to the relative powers of Nature or Art to cure Disease, is one that involves a variety of questions affecting the science, the practice, the polity, and the ethics of medicine. The regiminal treatment of disease is by no means an invention of modern date, but its merit, as opposed to a more decidedly medicinal treatment, divided the ancients, as it has formed a subject of discussion in more recent times. The public have also ever been alive to the question, in evidence of which we would only quote one lay-author, who, in the 'History of Foundling,' observes:

"If the number of those who recover by physic could be opposed to that of the martyrs to it, the former would rather exceed the latter. Nay, some are so cautious on this head, that to avoid the possibility of killing the patient, they abstain from all methods of curing, and prescribe nothing but what can neither do good nor harm."

Fielding evidently thought Nature was not to be trusted, for he continues:

"I have heard some of these with great gravity deliver it as a maxim, that Nature should be left to do her own work, while the physician stands by, as it were, to clap her on the back, and encourage her when she doth well."

The subject is one that every tyro in the profession occasionally reflects upon; but it is essentially one upon which the opinion of men who rank highest in the profession, by the length of their experience, or the labors by which they have achieved distinction, deserves to be listened to with the most respectful attention. The book before us is the production of one who, after having been engaged in the practice of medicine for fifty years, feels, to employ his own words, that his profession has claims on him for much more than he has hitherto been able to give it, but that he is called upon now to communicate any information he may possess, if he is to communicate it at all.

"In doing so," he continues, "I cannot help being impressed with the feeling of solemnity which naturally accompanies any act that is to be the last of its kind. And in this mood I would fain regard the present work in the light of *A Legacy to my younger brethren*, which, slight as it is, may not be found altogether unworthy of their acceptance. I would indeed bequeath it in full confidence of its value, if I might reckon on its being received in the same way as the Legacy of the Pot of Gold in the fable was received by the rustic testator's sons. If my book—though, like the old man's vineyard, really containing in itself no gold—should only lead to the zealous cultivation of the subject of which it treats, the result could not fail to be of inestimable value to the cultivators. For, on the profounder, more critical, and purer study of Nature as manifested in disease, rest, in my judgment, the best hopes of improvement in the medical art; and to this study the spirit of my book may, at least, lead the way and give the initiative, if its actual contents are found of less importance."

The error which the author regards as the great taint of medical science, and which he combats throughout this book, is a want of trust in the powers of Nature to arrest the process of disease, and a consequent overweening faith in remedial agents as the sole means of cure. His main object is to endeavor to expose these misconceptions, so as to impress the minds of the younger and less prejudiced members of the profession with the truth and importance of the principles advocated, and also to prepare a work which might convey to educated members of the general public a juster knowledge of the real nature of disease, and the true characters and powers of the medical art. Dealing professedly in deductions of which the premises are supposed to be known to the reader, we fear that, however intelligible the author may render his views to professional readers, the unprofessional reader will fail to appreciate them, because the first elements—the actual observation of disease—will be wanting to enable him to judge of the justness of Sir John Forbes's remarks. The current theories of the day generally ooze out sufficiently to infect the lay public; a true theory of disease, one, to use Mr. Spencer's words, of which the negation would be both false and inconceivable, once established among our Asklepiadai, and the public would not fail speedily to be imbued with so much knowledge as would be good for them. Until our own banquet is secured, we do better not to invite strangers to the feast; but we may prepare them against the happy consummation, by placing before them such sound and wholsome fare as they can safely digest. Let the public, high and low, be instructed in the plain, well-improved facts of anatomy and physiology; let them learn the wonders of our healthy fabric, and the greater marvels of the functions that minister to life; and thus prepared, we will induct them into the inner shrine, when we ourselves have raised the veil. Our duties to ourselves and to our high calling demand an humble, earnest, and assiduous devotion to the study of Nature in health and disease; we may thus ultimately succeed in understanding, to the full of man's capacity, workings that are yet mysterious; we may thus obtain, at last, what is the very starting point of Sir John Forbes' Theory of Therapeutics—*A Natural History of Disease.*

But, though we may be a long way from the goal, we must not ignore the fact that the whole tendency of the present age is to arrive at an interpretation of disease, such as the author shadows forth; Physiological Pathology, which forms the title of more than one work of distinction of recent date, has for its object the search into the relation of the morbid processes of disease to the analogous process of health; or into the mode in which the normal changes of the body are perverted, in the disturbances constituting a deviation from health. What is the meaning of the term metamorphosis, of tissues, now so often employed? Is it not applied equally in physiological and in pathological conditions, and do we not know that that metamorphosis passes through an endless variety of phases, both in health and disease; but that the transition is often imperceptible, showing the non-essentiality of disease as a separate entity, and proving that, in many cases at least, the same elements go to make up the sum of disease that previously, in a somewhat different order, constituted the normal state of health?

Let us suppose, for instance, that one of the essential features of pneumonia consists in the arrest of the chlorides of the blood in the lungs; physiology instructs us that a certain average of chlorides passes off by the urine in the four-and-twenty hours, and pathology informs us that an individual laboring under certain symptoms, termed inflammation of the lung tissue, the renal secretion yields a much smaller quantity of this constituent; we conclude that by restoring the secretion of chlorides by the kidneys, we shall relieve the organs of respiration, and promote the restoration of the various disturbed functions to their healthy condition.

The practical question, then, comes to be, whether we may or may not rely exclusively upon the natural powers to aid in its re-establishment, or whether we may trust Nature herself to effect it.

Again, let us take the case of a person wasting under a tubercular disease, in which hectic and colliquative sweats and diarrhœa are sapping the vital powers; we know that the metamorphosis of the tissues is going on so rapidly that, if left to herself, Nature will speedily wear out that thinning frame,—may we still place our faith in the tendency of disease to overcome its own evil, or in the unaided physiological powers to cast out the demon? or may we come to her aid with such helps as the study of Nature has proved to be conducive to an arrest of that excessive metamorphosis, and thus, if not save life, yet prolong its span? Or, to select a still more striking example, as capable of more positive synthetic and analytical proof:—in metalic poisoning, where all the tissues of the body are impregnated with the metal, and where disease is produced by the presence of the foreign substance in the muscles, the brain, the bones, what results are certain to occur if Nature, unaided, is required to cure the disturbance of function, the disease, that ensues? Will Nature eliminate the lead that causes the Burtonian line, or does she not rather wait for Art to come in with her sulphur or her iodide of potassium, to expel the enemy, who had effected so secure a lodgment that she alone would never have mastered him?

These are some of numerous instances that may be already alledged as indicative of the means by which we may rationally aid Nature in the treatment of disease; while especially the last two are brought forward to show that nature, or, in other words, the physiological forces of the system, do not suffice to restore health to the sufferer. We look forward with confidence to the time when the range of Art will be still further increased, and her relation to Nature still more accurately defined. But of one thing we are as certain as Sir John Forbes could desire us to be, that unless, in all our remedial administrations, we consult the laws of physiology, and seek to avail ourselves of the inherent powers of the constitution to remove disease, we shall ever fail to be masters of our art, and shall go about, ourselves blind, seeking to direct the blind. The real question in therapeutics is, what can Nature do unaided?—can she do it in the most expeditious way alone?—does she require some artificial assistance, or is she altogether unable to restore health without the intervention of art? In the above instance we have sought to illustrate some of the ways by which we may assist nature. Undoubtedly, in the majority of diseases, Nature, that is, the physiological process—tend to remove disease by restoring the natural balance of the powers; in many she does so most expeditiously, if left to herself; in many the processes may be facilitated if we adopt the hints which the balance and the test-tube afford; in some she appears overcome by the noxious influence, and to succumb, unless a powerful antidote be administered, which, by neutralizing the poison, enables the physiological influences again to assert their power, and to restore the balance of health.

We are tempted to place before the reader a formula by which we have sought to make clear to ourselves and to students the various points of view under which we may regard diseased processes, and which has appeared to us to facilitate the *rationale* of our therapeutical applications.

If we represent the normal constituents of the body by *a, b, c, d, e*, and assume that in health they occupy the relation to one another of *a*-|-*b*-|-*c*-|-*d*-|-*e*, disease may be regarded as taking place in one of three ways, which may as readily be represented by an algebraic formula.—Either the constituents are simply deranged, and they come to occupy a different mutual relation; or an elision of one or more of the elements may take place; or thirdly, a foreign element may be superadded. In the first case the different forms of disease may be as numerous as the changes that can be effected in the relative position of the elements, and instead of the formula, *a*-|-*b*-|-*c*-|-*d*-|-*e*, we may find them in the relation of *a*-|-*d*-|-*e*-|-*b*-|-*c*, or of *b*-|-*d*-|-*c*-|-*e*-|-*a*, and in many others. It is manifest that, if anywhere in the treatment of disease, this category will probably be the one in which we shall find the most numerous instances of what Sir John Forbes terms the power of Nature in curing disease,—that is to say, that the interference of medicinal agents will be less necessary, because, by placing the patient in a proper condition as regards noxious influences, by allowing the powers of his constitution to find their proper balance, the derangement of the constituents of health will be rectified. It becomes a question for further inquiry, whether it is not a due and legitimate prerogative of Art to ascertain that Nature is able to achieve certain results, when the conditions are granted, and to grant those conditions. We are willing at once to confess that since our first initiation into the mysteries of medicine we have abhorred the doctrine that our art consisted exclusively in the administration of pills and potions. To return.

Our second category—that in which an elision of one or more of the elements occurs—would be represented thus, $b + c + d + e$, or $a + b + e$, or let $E$ represent the elements in their normal strength and order, the above two instances would stand thus respectively: $E - a$, and $E - (c + d)$. The inquiry to be made in this case in regard to therapeutics would be, whether the powers of Nature would be adequate to restore the deficiency, or whether it would be requisite to supply the element which we found wanting by artificial means, or, in other words, by drugs. There can be no doubt that here again the physician will often do little more than place Nature in a position to work out her own salvation; but at the same time he may, by the judicious administration of the deficient element, materially expedite the process. To take a familiar instance. The blanched lip and the pale tongue of an anæmic patient challenges us to prescribe a salt of iron, because we know that element to be wanting to restore our patient to health. By hygienic means alone, by fresh air, improved diet, and cold sponging, the system may be enabled to take up from the food the necessary iron without medicines; but we all know that either we cannot place our patient in circumstances in which the hygienic means are available, or that the process is very tedious, and will be much expedited by the administration of five grains of the ammonio-citrate of iron three times daily, half-an-hour after meals. Then, naturally, both the patient and the physician agree that the citrate of iron shall be taken.

In our third category we place those forms of disease in which we discover a new element superadded to the normal constituents. This can be nothing else than a poison, which may be of an organic or an inorganic character. The formula here would be $(a + b + c + d + e +) + x$ or $+ y$; or, representing the normal elements collectively as $E$, the formula would stand $E + x$ or $+ y$. The duty of the medical man here would manifestly be first to recognize the presence of the $x$ or $y$, and then to determine upon its best mode of elimination. The same question arises as was put before. Do the powers of Nature suffice to produce the desired effect, or do they not? No one, we think, will affirm generally that they suffice; while probably every medical man of experience will readily admit that in many instances the unassisted powers of Nature will secure the elimination of the $x$ or the $y$. But we claim for the medical man the right to place Nature in the right position to develop her own powers, and we all know that the intervention of the physician for that purpose is often most essential, as Nature does not herself always assert her right to be heard in her own cause. Were it otherwise, probably neither Sir John Forbes' book nor this Review would have been written. On the other hand, it is unnecessary to quote instances in proof of the statement that unaided Nature—i.e., Nature without physic, *sensu strictiore*—is often inadequate to secure the elimination of $x$ or $y$, and that without the eliminant, $x$ or $y$ will continue to infest our patient's tissues, racking his nerves, exhausting his vigor, turning his muscles into fat. There may be a medium between these two extremes; we may know that Nature will suffice to remove the poison, but we know also that in its passage through the system it will leave an idelible impression, or that much time will be wasted by the process; and we have an agent at hand which will accelerate the movement of the elements, and secure a more rapid evolution of $x$ or $y$, shall we therefore not employ it because Nature might have carried out the work alone? Not to allow the employment of the drug under such circumstances would be tantamount to a wilful rejection of the boons accorded to us by Him from whom Nature has her source. *Omne simile claudicat*, and a limp may probably be discovered in any and every theory that was ever proposed; therefore, we guard ourselves against the charge of representing all diseased actions by the above formulæ. Before concluding this brief exposition, we would merely add, for the benefit of any one who may be tempted to follow out our method more into detail, that the three categories would, even theoretically, necessarily often pass into one another; thus, whether an element were deficient, or a new one superadded, in either case the remaining elements might change places in a manner represented by the first category; or an element might be wanting, while a foreign constituent were superadded; it is clear that by going into a detailed comparison between individual forms of disease and the formulæ, we should often meet with very complex arrangements; these we have at present nothing to do with, as it is not our wish to dilate upon a mathematical representation of disease, but simply to show the point of view in which we regard the various demands made by diseased processes upon the therapeutical interference of medicine.

We think that by the above analysis we have rendered to ourselves more clear what may be expected of Nature in Sir John Forbes' sense, what of Art. Still, before proceeding to a further examination of Sir John's views, we would protest against that assumed antithesis being made a ground of accusation against the medical profession at large.—Many there undoubtedly are who think that without medicine no disease can be well cured; but we feel satisfied that the great bulk of all educated members of the profession—and, thanks to modern progress, we may hope that that comprises the majority of all its members—consider themselves rather as ministers and interpreters of Nature than as Titans who are engaged in a constant warfare with her vicious manifestations. That interpretation may often be erroneous, but the attempt to discover it argues for the fact that the medical man does not desire to place his art in contrast with Nature.

If we are right in assuming these remarks to apply very generally to medical men of the present day, we by no means assert that such tendencies have always prevailed; the fact that they did not prevail some years ago, in this country at least, was proved by the animated opposition which the enlightened views of Sir John (then Dr.) Forbes excited when put forth in 1845, in an article on Homœopathy, Allopathy, and Young Physic. Twelve years are but a comparatively brief space of time in the development of a nation, or of an important integral part of a nation, yet any one who is conversant with the feelings, and views, and the general state of education of medical men, during the last twenty years, will, we think, bear us out in the opinion that, scientifically and ethically, great onward strides have been made. We cannot but believe that the courageous advocacy by Sir John Forbes of views much opposed to the prejudices of a large class in the profession, have contributed not a little towards the reformation of the *drugging system.* Most sincerely do we thank him as a benefactor of his profession and of mankind. At the same time we are constrained to admit that we are far from arriving at that goal of certainty which we all desire to attain; and we would willingly enlarge our knowledge of the relative powers of Nature and Art in the cure of disease. Sir John appreciates the difficulties that impede the attainment of this desirable end, but speaks more lightly of them than we think the case justifies.

"The main obstacles . . . . lie rather in the circumstances under which the subjects are presented to them than in the subjects themselves. When we have the proper field for investigation before us, there is very little difficulty in obtaining a positive and accurate knowledge of the power possessed by Nature in relieving and curing diseases. The phenomena to be observed are neither very numerous nor very complex; the facts are easily obtained, and the deductions are both facile and sure. All that is requisite to insure a positive and pure result is, in the first place, to take care that no artificial interference disturbs the organic processes going on; and, in the second place, to observe and chronicle progressive events. It is a case of simple observation throughout; no sifting of premises, no elimination of causes, no grouping or balancing of effects being requisite to insure a just conclusion. The just conclusion—the exact valuation or appreciation of the power under examination—is enunciated in the simple fact indicating what has been the issue of the organic processes constituting the disease. The sum total of beneficial modification of the morbid processes, whatever it may be, whether amounting to a complete or an imperfect cure, must be acknowledged to be the exclusive work of Nature; in other words, of the conservative powers inherent in the living."—(p. 25.)

The author admits that there are difficulties in finding the proper field for study; but this we do not regard as so serious a difficulty as the determination of where medicinal interference commences, and where it ceases. All writers on materia medica claim baths, hot and cold, as belonging to the *armamentarium medicorum;* the regiminal physician would probably claim them as coming within the pale of regiminal treatment, though we should be inclined to assert that the natural man abhors the cold bath, and only learns to love it by degrees. The application of ice externally and internally is a powerful remedy, but may belong to either party; poultices to the surface or the mucous membranes (in the shape of mucilaginous beverages) are instances of a similar kind; ripe fruits, raw or cooked, are regiminal, but their acids and neutral salts administered in solution would be eschewed, because they would come to the house by aid of the apothecary's boy. We quote these instances in no spirit of levity, but to show that the question is not in reality so much one to be settled by two opposing parties—the medicinal and the regiminal physicians—but that the inquiry to be prosecuted must and may be carried out contemporaneously by all who are anxious to elevate the science of medicine, and place it on a sure basis. No humane physician would consent to watch the progress of a case of neuralgia under purely regiminal treatment, when he knows that a grain of morphia will arrest the pain and give rest to the sufferer; nor would he allow a patient's health to continue to deteriorate because he did not choose to administer a drachm of the oil of male fern for the removal of tænia which infest his intestine. These, and numerous other instances of a similar character, would have to be eliminated before fixing either the diseases or the drugs which were to form the subject of study. Thus the question necessarily becomes more and more narrowed, and at last we arrive at the conclusion that what we have to investigate is rather how much or how little influence individual drugs are able to exert upon certain diseases than that we declare ourselves followers of this or that banner. When we examine the author's statements with regard to the "instruments of the medical art," we gather that, although so powerful an advocate of an essentially regiminal [illegible]eatment of disease, he by no means despises or rejects the exhibition of drugs; thus, in speaking of alteratives, he says, "the class contains some remedies of positive and evident power;" he lauds opium and its products, as being "some of the noblest instruments of the medical art," and says that "the class contains a good many other agents of analogous though inferior value." Of genetica he says that the "class contains only three or four drugs, but they possess a positive power of greater or less extent." Scarcely a class is passed in review of which not something more or less favorable is said; we doubt much whether a physician who believes—may we not say knows?—that he has command of a remedy of "obvious and admirable power," would refuse to allow his patient all the relief the drug can afford, in order that he might test whether the unaided powers of Nature sufficed to remove the disease he labors under.

The reader will naturally inquire into the manner in which the author of the book under consideration reconciles the apparent contradiction involved in his all but unlimited faith in the powers of Nature to cure disease, on the one hand, and his laudation of drugs, as exhibited in our last remarks, on the other. We will quote his own words in reply, merely premising that Sir John terms the system he advocates, *Auxiliary or mild treatment, rational expectancy.*

"This modification of the indirect physiological method of treating diseases (more especially acute diseases,) I regard as at once the most philosophical, the safest, the surest, and the most successful of all the forms it assumes. . . . . In the first place, it completely recognizes the autocracy of nature in the cure of acute diseases, and proceeds on the principle that it is not only useless, but injurious, to attempt to suppress or greatly to modify the morbid processes by strong measures of a perturbative or exhaustive kind.

"The indications which this mode of treatment seeks to fulfil are chiefly the following:—1st. To place the diseased body in the most favorable circumstances for the development and exercise of its own conservative powers, by the institution of a proper regimen, in the most comprehensive sense of that term. 2d. To endeavor thereby, or through the use of medicaments, to remove such obstacles to the favorable action of the conservative and restorative powers as may be removable without the risk of checking or injuriously perverting them. 3d. Applying these measures under a watchful supervision; not to attempt by any vigorous measures to alter the course of the morbid processes so long as they seem to keep within the limit of safety, and when they transgress, or threaten to transgress this limit, only then to endeavor to modify them by such mild measures as, if they fail in doing good, cannot do much harm. 4th.

To be on the watch against possible contingencies, which may demand the employment of measures of exceptional activity, whether in the form of regimen or medicine, and, when required, to apply such measures with the necessary vigor.''—(p. 239.)

So far as this method combats the purely empirical proceedings and the treatment *a la Sangrado* that we all have seen, we most heartily concur with Sir John Forbes, and we think that if he had much familiar intercourse with physicians of less than twenty years' standing, and had the opportunity of judging of their practice, he would find that the prevailing fault is not so much a tendency to trust over much in drugs, as to be sceptical of their utility. There is a scepticism which leaves its holder in a slough of despond—a scepticism which, having no positive basis whatever, vacillates with the wind of public opinion or with the accidents of daily life. The medical man whose knowledge is of a calibre to allow him to become the prey of such scepticism, may be regarded as the most unfortunate man under the sun, for no professional act of his can be attended with any degree of moral satisfaction; he will be inclined to say with Dr. Cayol, " Les systemes en medicine sont les idoles auxquelles on sacrifie des victimes humaines." But there is another scepticism, which is the characteristic of all men of science, which co-exists with the warmest love of his profession and a full faith in the powers of his art, in the heart of the zealous and earnest physician ; it is the skepticism which leads him to be suspicious of his own observations and of his deductions, until by repetition and by comparison with the results obtained by others, he has placed his conclusions on the firmest basis upon which a scientific fact can rest. Such is the scepticism which has ever distinguished the most elevated in our ranks,--we think that the general advancement of the profession, and the greater humility which that increased knowledge has brought with it, have spread more generally that form of scepticism which we would uphold as a laudable feature of our times. Still we are also satisfied that while many of the views advocated by Sir John Forbes are in a great measure the views held by the majority of physicians of the present day, we have a more positive knowledge of the extent and limits of the powers of Nature on the one hand, and of the real uses of drugs on the other, in the cure of morbid states, than has been possessed by our predecessors. If we keep to the path we are pursuing, and continue to seek to interpret Nature correctly in all her phases, making Physiology our main instructress in the manifestations of disease, but not disclaiming any aid obtainable from all the handmaidens of the physician, we shall pass safely through the Scylla and Charybdis of dogmatism and false scepticism. We shall retain our faith in the powers of Nature to cure disease, but we shall no less continue to believe that "the Lord hath created medicines out of the earth, and he that is wise will not abhor them."

In the preceding remarks we have sought rather to indicate the spirit that pervades Sir John Forbes' book than to give our readers a summary of its contents. With its spirit we cordially sympathize, for it is a spirit of earnest hatred of all quackery, of manly affection for the high objects of our common profession. We cannot deny that the author appears to us to underrate the value of medicines, but so long as with us the professional man and the tradesman are blended together, and Government takes quackery under its special protection, so long there will be little risk of drugs falling generally into disrepute; and we may say of Sir John, as of the Man of Ross, that

"E'en his errors leant to virtue's side."

With this reservation, we do not hesitate to repeat that we cordially agree with the author's general views, and recommend his "Legacy" to all medical men who earnestly seek for truth in the daily practice of their surpassingly interesting profession.

